# Inhibition of SIRT2 by Targeting GSK3β-Mediated Phosphorylation Alleviates SIRT2 Toxicity in SH-SY5Y Cells

**DOI:** 10.3389/fncel.2019.00148

**Published:** 2019-04-24

**Authors:** Shuhu Liu, Zhihua Zhou, Ling Zhang, Siying Meng, Shuji Li, Xuemin Wang

**Affiliations:** Key Laboratory of Mental Health of the Ministry of Education, Guangdong Province Key Laboratory of Psychiatric Disorders, Department of Neurobiology, School of Basic Medical Sciences, Southern Medical University, Guangzhou, China

**Keywords:** SIRT2, GSK3β, 6-OHDA, PD, neuroprotection

## Abstract

Sirtuin 2 (SIRT2) is thought to be important in the pathogenesis of Parkinson’s disease (PD), and the inhibition of SIRT2 rescues α-synuclein toxicity in a cellular model of PD. Recent studies have focused on identifying inhibitors of SIRT2, but little is known about the processes that directly regulate its function. GSK3β is a serine/threonine protein kinase that affects a wide range of biological functions, and it is localized in Lewy bodies (LBs). Therefore, we investigated whether SIRT2 is regulated by GSK3β and enhances cell death in PD. In the present study, Western blot showed that total SIRT2 levels did not change noticeably in a cellular model of PD but that SIRT2 phosphorylation was increased, and GSK3β activity was elevated. In addition, mass spectrometry (MS) studies indicated that SIRT2 was phosphorylated by GSK3β at three specific sites. Phospho- or dephospho-mimicking studies demonstrated that this postmodification (phosphorylation) increased SIRT2 toxicity in SH-SY5Y cells. Collectively, our findings identify a posttranslational mechanism that controls SIRT2 function in PD and provide evidence for a novel regulatory pathway involving GSK3β, SIRT2, and α-synuclein.

## Introduction

Parkinson’s disease (PD) is a long-term neurodegenerative disorder that mainly affects the motor system. Since it was first reported by James Parkinson in 1817 (Lees, 2007), PD has become the second most common neurodegenerative disorder (Dawson and Dawson, 2003). The disease is characterized by the loss of dopaminergic neurons in the substantia nigra, and the pathological hallmark is the accumulation of α-synuclein protein aggregates called Lewy bodies (LBs). It is believed that the number and size of α-synuclein aggregates affect the progression of PD. However, the exact molecular mechanisms that contribute to dopaminergic neuron loss remain to be clarified.

To explore the molecular mechanisms of PD, we focused on sirtuins, a family of class III nicotinamide adenine dinucleotide-dependent deacetylases that deacetylate histone and nonhistone proteins. SIRT2 is one of seven members of the sirtuin family, and it plays diverse roles in cellular metabolism and aging. SIRT2 is highly expressed in the central nervous system (Maxwell et al., 2011; Zhu et al., 2012) and is located in the cytoplasm (North et al., 2003), the nucleus (Dryden et al., 2003; North et al., 2003) and mitochondria (Liu et al., 2017). It has been reported that SIRT2 plays essential roles in PD, and SIRT2 inhibitors rescue α-synuclein-mediated toxicity (Outeiro et al., 2007; Chen et al., 2015). One SIRT2 inhibitor, AK7, even protects against MPTP neurotoxicity in mice (Chen et al., 2015). The protective effects of SIRT2 inhibition are mediated through a number of pathways. First, SIRT2 can directly deacetylate α-synuclein to exacerbate α-synuclein toxicity *in vivo* (de Oliveira et al., 2017). Second, SIRT2 inhibition achieves neuroprotection by reducing sterol levels *via* the decreased nuclear trafficking of SREBP-2 (Luthi-Carter et al., 2010). Third, SIRT2 inhibition may be neuroprotective in PD by modulating a redox network (Wang et al., 2015; Guan et al., 2016). Although SIRT2 plays a key role in the development of PD, we still do not know how SIRT2 itself is regulated during the development of this disease. It has been reported that SIRT2 is a phosphorylation substrate of CDK5, which modulates the activity of SIRT2 (Pandithage et al., 2008). However, there have been no reports that CDK5 can regulate the activity of SIRT2 in PD. To obtain further insight into the mechanism by which SIRT2 is regulated, we sought to identify novel upstream kinases of SIRT2. GSK3β and CDK5 are two kinases at the center of research on Alzheimer’s disease, and they share the same substrate (Wen et al., 2008). Therefore, we hypothesized that SIRT2 may be a substrate of GSK3β.

GSK3β is a serine/threonine protein kinase that is activated by neurotoxins (Hongo et al., 2012; Hernandez-Baltazar et al., 2013; Zhao et al., 2016) and PD-associated gene mutations (Wang et al., 2013; Kawakami et al., 2014). Additionally, in the postmortem PD brain, GSK3β is localized in LBs, as is phosphorylated GSK3β (Ser9; Nagao and Hayashi, 2009). Furthermore, in a study of a group of 251 Spanish patients with PD, Infante et al. (2010) found that a GSK3β (rs6438552) TT genotype, which has been shown to produce a more active isoform (Kwok et al., 2005), is associated with an elevated risk of PD. Thus, GSK3β is important in the development of PD. In accordance with these reports, GSK3β downregulation partially abrogates 6-OHDA-induced SH-SY5Y apoptotic cell death (Li et al., 2011) and MPP (+)-induced neuronal death (Petit-Paitel et al., 2009). These results indicate that GSK3β is a critical mediator of 6-OHDA/MPP (+)-induced neurotoxicity.

Based on the above information, we propose that SIRT2 may be phosphorylated by GSK3β during the development of PD. Here, we provide detailed insight into the mechanism through which GSK3β modulates SIRT2 activity and suggest that the phosphorylation of S327, S331 and S335 may be useful as a target for therapeutic intervention in PD.

## Materials and Methods

### Materials

An MTT assay kit was purchased from Roche. A site-directed mutagenesis kit was purchased from Stratagene. 6-Hydroxydopamine hydrobromide (6-OHDA), DMSO, SB216763 (S3442, an inhibitor of GSK3β) and AGK2 (A8231, an inhibitor of SIRT2) were obtained from Sigma-Aldrich. Antibodies against pGSK3β (Ser9) and GSK3β were purchased from Cell Signaling (Danvers, MA, USA). Antibodies against SIRT2, ace-tubulin, α-tubulin, HA and Flag were purchased from Sigma-Aldrich. Secondary antibodies conjugated to Alexa 488 or Alexa 594 were purchased from Invitrogen. Hoechst 33258 (94403) was purchased from Sigma-Aldrich. Protein A/G-coated Sepharose beads were obtained from Santa Cruz Biotechnology (Dallas, TX, USA). An anti-phosphoserine/threonine/tyrosine antibody was obtained from Abcam (ab15556). Protein kinase CDK5/p25 (cat. 14—516) and GSK3β (cat. 14—306) were purchased from Millipore. Cells were transfected using Lipofectamine 2000 Transfection Reagent (Invitrogen, Thermo Fisher Scientific, Waltham, MA, USA). Other chemicals and reagents were of the highest analytical grade and were purchased from local commercial sources.

### Cell Culture

The human neuroblastoma cell line SH-SY5Y was obtained from the American Type Culture Collection. The cells were grown in Dulbecco’s modified Eagle’s medium (DMEM)/Ham’s F12 (1:1 mixture; HyClone) supplemented with 10% fetal bovine serum (GIBCO) in a 5% CO_2_ incubator at 37°C. Human embryonic kidney cells (HEK293) were grown in DMEM (HyClone) supplemented with 10% FBS.

### Pharmacological Treatments

6-OHDA was dissolved in phosphate-buffered saline (PBS) and used at a final concentration of 100 μM (Ikeda et al., 2008), which was the dose shown to induce 50% cell death within 24 h after 4 h of exposure. SB216763 and AGK2 were dissolved in DMSO. Before adding 6-OHDA, the SH-SY5Y cells were treated with SB216763 (Acevedo et al., 2014) or AGK2 (Outeiro et al., 2007) at a final concentration of 10 μM for 0.5 h (the DMSO content never exceeded 0.1%).

### MTT Tests for Cell Viability

Cell viability was measured by a colorimetric MTT [3-(4,5-dimethylthiazol-2-yl)-2,5-diphenyltetrazolium bromide] assay. Briefly, the cells were seeded in a 96-well plate at a density of 5 × 10^4^ cells/well. The cells were then pretreated with SB216763 for 0.5 h. After pretreatment, 6-OHDA was added to the culture medium to reach a final concentration of 100 μM. The control cells were not treated with SB216763 or 6-OHDA. The culture medium was changed after incubation for 4 h. Then, 16 h later, the MTT reagent was added to each well, and the cells were incubated for an additional 4 h. The absorbance of each reaction product was measured with a microplate reader at a wavelength of 595 nm. The results are expressed as a percentage of the MTT absorbance of the control cells, which was set to 100%.

### Western Blot Analysis

Whole-cell lysates were prepared by incubating cells in RIPA buffer supplemented with a protease inhibitor cocktail (Selleck) according to the manufacturer’s instructions. Briefly, the cells were harvested by centrifugation at 900 rpm for 5 min and washed in PBS (pH 7.2). The pellets were solubilized in the same volume of lysis buffer, kept on ice, vortexed for 5 min, and centrifuged at 13,000× *g* for 20 min at 4°C. Equal amounts of total lysate protein were loaded and separated on a 10% sodium dodecyl sulfate-polyacrylamide gel electrophoresis (SDS-PAGE) gel. The proteins were electrophoretically transferred to a PVDF membrane, and the membrane was blocked in 5% skim milk in Tris-buffered saline containing 0.1% Tween-20 (TBST) for 1 h. Then, the membranes were incubated at 4°C overnight in the presence of a primary antibody against one of the following proteins: HA, Flag, SIRT2, ace-tubulin, α-tubulin (Sigma), GSK3β or pGSK3β (Ser9). Next, the membranes were washed three times with TBST every 5 min and probed with a corresponding horseradish peroxidase (HRP)-conjugated secondary antibody at room temperature for 1 h. Probe detection was conducted using enhanced ECL Advance Western Blotting Detection Reagents (Perkin Elmer, Waltham, MA, USA) and a ChemiDoc XRS+ system (Bio-Rad, Hercules, CA, USA).

### Immunofluorescence Staining

Cultured cells were fixed with PBS containing 4% paraformaldehyde for 15 min, which was followed by washing and permeabilization in PBS with 0.1% Triton X-100 for 5 min. The samples were blocked in PBS with 5% BSA and incubated with primary antibodies overnight at 4°C. The following primary antibodies were used: rabbit anti-SIRT2 (1:100) and mouse anti-acetylated α-tubulin (1:50). Secondary antibodies conjugated to Alexa 488 or Alexa 594 (1:500) were applied for 1 h at room temperature. After washing with PBS and staining with DAPI for 5 min, coverslips were mounted with Mowiol. Images of the cells were acquired at room temperature on a confocal microscope (A1+; Nikon) using either a 40× or a 60× lens. Photoshop CS (Adobe) was used to adjust the contrast and brightness.

### Coimmunoprecipitation

Cells were collected and lysed in buffer containing 50 mM Tris-HCl at pH 7.4, 150 mM NaCl, and 1% NP-40. All buffers were supplemented with protease and phosphatase inhibitors. The extracted proteins were precleared by incubating the lysates with Protein A/G-coated Sepharose beads for 1 h at 4°C, and then the supernatants (500 μg, 1 μg/μl) were incubated overnight with an HA or Flag antibody at 4°C. Precipitation of the immune complexes was performed with Protein A/G-coated Sepharose beads for 2 h at 4°C. After immunoprecipitation, the beads were washed five times with lysis buffer at 4°C and suspended in 40 μl of 2× loading buffer. All samples were loaded onto a gel, processed by SDS–PAGE and analyzed by Western blot. To detect SIRT2 phosphorylation, we used an anti-phosphoserine/threonine/tyrosine antibody.

### Plasmids and Transfection

GSK3β-HA and SIRT2-flag were obtained from Addgene. PCMV-HA and PCMV-Tag4A are blank plasmids that contain an HA tag and a Flag tag, respectively. A subsequent LR-recombination reaction with PGEX4T3 and phMGFP plasmids resulted in GST-tagged and GFP-tagged expression constructs. Site-directed mutagenesis was performed using a Quick change protocol to generate the S327A, S331A, S335A, S327&331&335A and S327&331&335D mutants. All constructs were verified by DNA sequencing. SIRT2-targeting shRNAs using previously identified sequences (Si et al., 2013) were cloned into a pGPU6/GFP/Neo vector from Genepharma. For transient transfection, cells were transfected using Lipofectamine 2000 Transfection Reagent according to the manufacturer’s instructions.

### Determining the Survival Rate of Transfected Cells

SH-SY5Y cells were transfected with plasmids (GFP, GFP-SIRT2, GFP-SIRT2SA or GFP-SIRT2SD) for 24 or 48 h to induce the expression of GFP. Chromatin condensation was detected by nuclear staining with Hoechst 33258. Briefly, the cells were fixed with 4% paraformaldehyde for 15 min, stained with PBS/0.1% TritonX-100/10 μM Hoechst 33258 for 5 min, and then visualized by fluorescence microscopy. Apoptotic cells were stained bright blue because of their chromatin condensation. Images were captured from different fields of each well for different groups of cells at 20× magnification using an Olympus fluorescence microscope. The numbers of total and live transfected cells were counted from multiple fields to obtain the percentage of live transfected cells (Dutta et al., 2018).

### GST Fusion Protein Purification

The GST-SIRT2 expression plasmid was transformed into competent *Escherichia coli* BL21 cells. A single colony was inoculated in LB medium at 37°C in an orbital shaker incubator until the mid-log phase. IPTG (100 μM) was added to the culture and incubated at 20°C for 8 h. Following the incubation, the culture was centrifuged at 2,500× *g* for 10 min at 4°C, and the resulting pellet was resuspended in binding buffer [50 mM Tris-Cl, pH 8.0, 150 mM NaCl and 1× protease inhibitor cocktail (Biotool)]. The cells were lysed by sonication and centrifuged at 12,000× *g* for 30 min at 4°C. After centrifugation, the supernatant was collected, incubated with GST agarose beads (Novagen) and maintained under agitation overnight at 4°C. After overnight binding, the beads were washed with wash buffer (150 mM NaCl, 50 mM Tris-Cl, pH 7.5 and 1% Triton X-100), and the bead-bound proteins were eluted and stored in 30% glycerol-containing buffer. Other GST fusion proteins were obtained in the same way.

### *In vitro* Kinase Assays

The phosphorylation of GST-SIRT2 was performed in a final volume of 25 μl consisting of 5 mM MOPS, pH 7.2, 0.05 mM DTT, 4 mM MgCl_2_, 80 μm ATP, 10 μCi of [γ-32P]ATP, 0.4 mM EDTA, 1 mM EGTA, and 2.5 mM β-glycerophosphate for 30 min at 30°C in the presence or absence of CDK5/p25 or GSK3β. The reaction was terminated by the addition of 25 μl of 2× sodium dodecyl sulfate (SDS) loading buffer (187 mm Tris-HCl, pH 6.8, 30% (w/v) glycerol, 6% SDS and 15% β-mercaptoethanol). The samples were then heated at 100°C for 5 min, centrifuged in a microcentrifuge, and loaded on a gel for SDS-PAGE. Following electrophoresis, the proteins were exposed to a Kodak X-ray film for autoradiography at −80°C for 2 or 3 days.

### Phosphopeptide Identification by Mass Spectrometry (MS)

After *in vitro* phosphorylation, GST-SIRT2 was separated *via* SDS-PAGE. The gel was cut out after Coomassie blue staining, and the samples were sent to the Beijing Proteome Research Center (BPRC, China).

### Statistics and Reproducibility

Statistical analysis was performed using Student’s two-tailed unpaired *t*-tests for two-group comparisons and one-way analysis of variance (ANOVA) or two-way ANOVA for multigroup comparisons. *P* values < 0.05 were considered statistically significant and are indicated with asterisks (*P < 0.05; **P < 0.01; ***P < 0.001; ****P < 0.0001) in the figure legends. All of the data collected met the normal distribution assumptions of the tests. The data are represented as the mean ± SD. Each experiment was performed at least three times and in duplicate or more.

## Results

### 6-OHDA Elevates the Phosphorylation of SIRT2 in SH-SY5Y Cells

**Figure 1:**
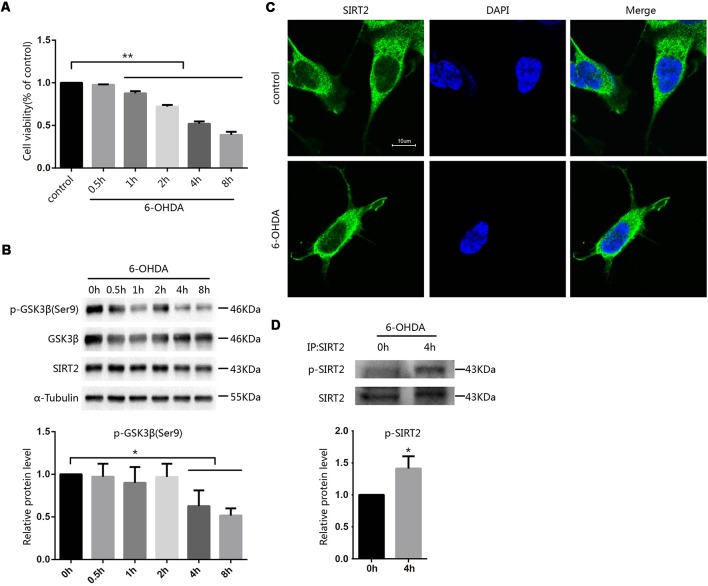
Phosphorylation of SIRT2 increased after treatment with 6-OHDA. **(A)** SH-SY5Y cells were treated with 100 μM 6-OHDA for the indicated time. After 24 h, cell viability was measured by the MTT assay [**p < 0.01, one**-**way analysis of variance (ANOVA) followed by Tukey’s *post hoc* test]. **(B)** Western blot analysis showing the phosphorylation of GSK3β at Ser9 and total GSK3β, SIRT2 and α-tubulin levels in SH-SY5Y cells treated with 100 μM 6-OHDA for the indicated time. GSK3β activity was measured by detecting the phosphorylation of GSK3β at Ser9 by Western blot (*p < 0.05, one-way ANOVA followed by Tukey’s *post hoc* test; **C**). Fluorescence images representing SIRT2 immunofluorescence in phosphate-buffered saline (PBS)/6-OHDA-treated SH-SY5Y cells. Scale bar: 10 μm. **(D)** Western blot analysis showing SIRT2 phosphorylation in cells. Endogenous SIRT2 was immunoprecipitated from SH-SY5Y cells treated with or without 100 μM 6-OHDA for 4 h and probed with an anti-phosphoserine/threonine/tyrosine antibody to detect phosphorylation levels (*p < 0.05, **p < 0.01, two-tailed Student’s *t*-test). The values are expressed as the mean ± SD, *n* ≥ 3 for each group.

To investigate how toxins regulate SIRT2 function, we first established a cellular model of PD by applying 100 μM 6-OHDA to SH-SY5Y cells at the indicated times and concentrations and then assessed cell viability by the MTT assay. Cell viability decreased in a time-dependent manner following 6-OHDA exposure (Figure 1A). Further, we evaluated the expression of SIRT2 in SH-SY5Y cells after treatment with 6-OHDA, and SIRT2 levels showed a slight but not noticeable decrease. Furthermore, the active status of GSK3β was increased (Figure 1B). Because SIRT2 can be distributed in the cytoplasm or nucleus under different conditions (Dryden et al., 2003; North et al., 2003; Liu et al., 2017), we wanted to know where SIRT2 is located under 6-OHDA treatment. We next treated SH-SY5Y cells with PBS as a control or 100 μM 6-OHDA for 4 h; later, the location of endogenous SIRT2 was assessed by immunofluorescence. We observed that SIRT2 resided in the cytoplasm for the duration of the experiment (Figure 1C). We next purified endogenous SIRT2 from SH-SY5Y cells treated with or without 6-OHDA and detected SIRT2 phosphorylation using an anti-phosphoserine/threonine/tyrosine antibody. We found that phosphorylated SIRT2 levels were significantly increased after treatment with 6-OHDA (Figure 1D).

### The Protective Effect of GSK3β Inhibition Occurs Through a Blockade of SIRT2 Phosphorylation

**Figure 2:**
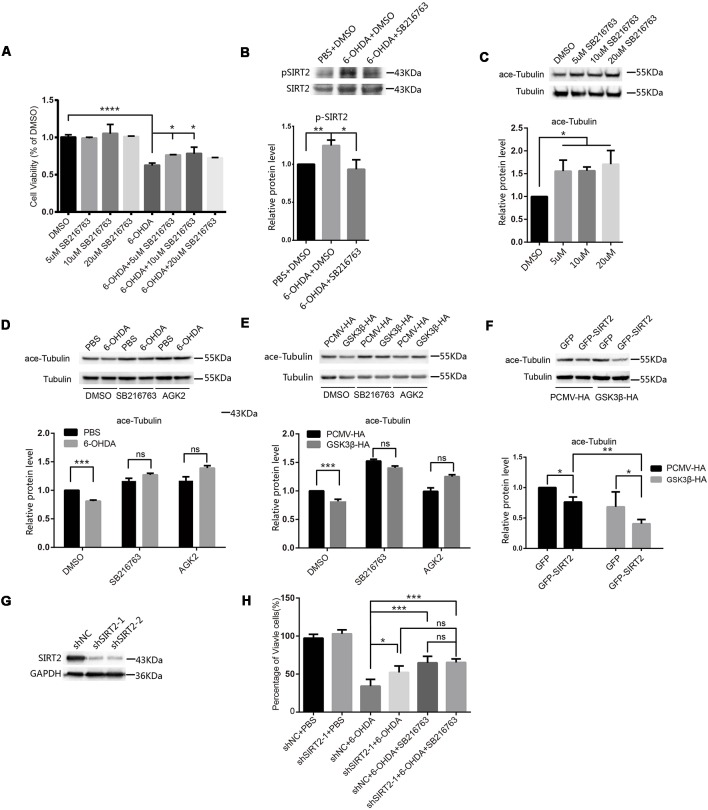
Inhibition of GSK3β protected SH-SY5Y cells from 6-OHDA by decreasing SIRT2 phosphorylation. **(A)** SH-SY5Y cells were pretreated with different concentrations of SB216763 for 30 min and then treated with or without 100 μM 6-OHDA for 4 h. Cell viability was measured by the MTT assay (*p < 0.05, ****p < 0.0001, one-way ANOVA followed by Dunnett’s *post hoc* test). **(B)** Endogenous SIRT2 was immunoprecipitated from whole-cell lysates, and p-SIRT2 was detected by an anti-phosphoserine/threonine/tyrosine antibody (*p < 0.05, **p < 0.01, one-way ANOVA followed by Tukey’s *post hoc* test). **(C)** Western blot analysis showing the acetylation of α-tubulin (ace-tubulin) and total α-tubulin levels in SH-SY5Y cells treated with DMSO or SB216763 at the indicated concentrations (*p < 0.05, one-way ANOVA followed by Dunnett’s *post hoc* test). **(D)** SH-SY5Y cells were pretreated with DMSO, 10 μM SB216763 or 10 μM AGK2 for 30 min and then treated with or without 100 μM 6-OHDA for 4 h. Ace-tubulin and α-tubulin were detected by Western blotting (***p < 0.001, two-way ANOVA followed by Bonferroni’s *post hoc* test). **(E)** HEK293 cells were treated with DMSO, 10 μM SB216763 or 10 μM AGK2 for 30 min after transfection with PCMV-HA as a control or GSK3β-HA for 24 h. Ace-tubulin and α-tubulin were detected by Western blotting (***p < 0.001, two-way ANOVA followed by Bonferroni’s *post hoc* test). **(F)** HEK293 cells were cotransfected with GFP (or GFP-SIRT2) and PCMV-HA (or GSK3β-HA) for 24 h. Ace-tubulin and α-tubulin were detected by Western blotting (*p < 0.05, **p < 0.01, two-way ANOVA followed by Tukey’s *post hoc* test). **(G)** The level of the SIRT2 protein was downregulated in SH-SY5Y cells transfected with SIRT2 shRNA (shSIRT2-1 or shSIRT2-2) compared to cells transfected with the control (shNC). SIRT2 and GAPDH were detected by Western blotting. **(H)** A histogram representing the percentage of viable cells. Cells were transfected with shRNA for 48 h and then treated with 6-OHDA for 4 h. The percentage of viable cells was calculated after 24 h (*p < 0.05, **p < 0.01, ns, not statistically significant; one-way ANOVA followed by Tukey’s *post hoc* test). The values are expressed as the mean ± SD, *n* ≥ 3 for each group.

Given that the activity of GSK3β was increased in SH-SY5Y cells treated with 6-OHDA, we blocked the activity of GSK3β in SH-SY5Y cells with SB216763 to determine whether this could protect the cells from 6-OHDA. We pretreated SH-SY5Y cells with different concentrations of SB216763 for 30 min and then applied 100 μM 6-OHDA for 4 h. After that, we measured cell viability and found that SB216763 rescued the SH-SY5Y cells from 6-OHDA (Figure 2A). We further analyzed the phosphorylation of endogenous SIRT2 among the control, 6-OHDA and 6-OHDA plus SB216763 groups and found that the phosphorylation of SIRT2 was increased in the 6-OHDA-treated cells, while SB216763 blocked the increase in SIRT2 phosphorylation (Figure 2B). These results indicate that GSK3β activation may be responsible for the increased phosphorylation of SIRT2.

SIRT2 is a tubulin deacetylase, and SB216763 increases the acetylation of tubulin (Figure 2C), which indicates that the decrease in SIRT2 phosphorylation mediated by SB216763 may reduce SIRT2 deacetylase activity. To further confirm this, we pretreated SH-SY5Y cells with DMSO, SB216763 or AGK2 and monitored the change in acetylated tubulin under 6-OHDA treatment. Acetylated tubulin levels decreased under 6-OHDA treatment, and both SB216763 and AGK2 reversed the decrease in acetylated tubulin levels (Figure 2D). To confirm that GSK3β participates in the regulation of SIRT2 deacetylase activity, we overexpressed PCMV-HA or GSK3β-HA in HEK293 cells and treated them with DMSO, SB216763 or AGK2 24 h later. The overexpression of GSK3β resulted in a decrease in acetylated tubulin levels, while SB216763 and AGK2 reversed the decrease in acetylated tubulin levels induced by the overexpression of GSK3β (Figure 2E). GSK3β further reduced acetylated tubulin levels relative to SIRT2 overexpression (the acetylated tubulin levels decreased by 23.73% and 40.65% in the absence or presence of GSK3β, respectively; Figure 2F). To further confirm that SIRT2 is the downstream target of GSK3β, we used shRNA to knock down endogenous SIRT2 (Figure 2G). We found that downregulated SIRT2 could reduce the toxicity of 6-OHDA, while SB216763 could not further improve cell viability after SIRT2 knockdown (Figure 2H). These data suggest that SIRT2 is the downstream target of GSK3β under 6-OHDA treatment. Taken together, our data demonstrate that 6-OHDA or the overexpression of GSK3β induces a decrease in acetylated tubulin levels and that inhibiting GSK3β with SB216763 counteracts this effect.

### GSK3β Can Phosphorylate SIRT2 at Three Particular Sites

**Figure 3:**
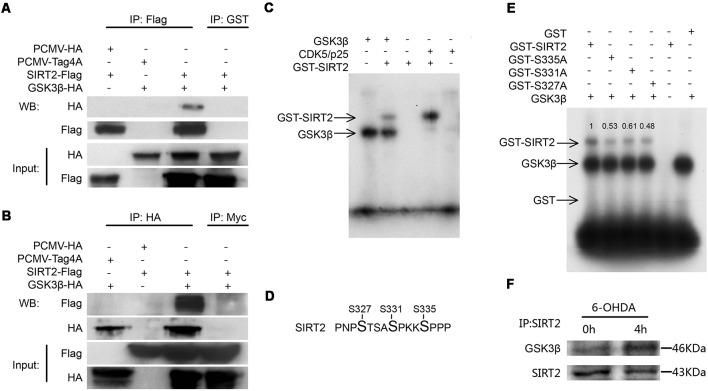
SIRT2 could be phosphorylated by GSK3β *in vitro*. Panels **(A,B)** show the immunoprecipitation and immunoblot analyses of the interaction between SIRT2 and GSK3β in HEK293 cells expressing Flag-tagged SIRT2 or HA-tagged GSK3β. Anti-GST and anti-Myc antibodies were used as the IgG controls. **(C)** GST-SIRT2 was purified from BL21 *E. coli*, and an *in vitro* kinase assay was performed with or without GSK3β or CDK5/p25. GST-SIRT2 phosphorylation was confirmed by autoradiography. Because GSK3β is able to autophosphorylate, GSK3β bands are visible. **(D)** An *in vitro* kinase assay was performed with or without GSK3β. The samples were subjected to mass spectrometry (MS) to detect the phosphorylation of SIRT2. The S327, S331 and S335 sites were phosphorylated. **(E)** Serine was genetically mutated to alanine to mimic the dephosphorylation of the S327, S331 and S335 sites; then, the *in vitro* phosphorylation of these mutants by GSK3β was detected with autoradiography. **(F)** Endogenous SIRT2 was immunoprecipitated from SH-SY5Y cells treated with or without 100 μM 6-OHDA for 4 h. Western blotting analysis showing that the interaction between SIRT2 and GSK3β increased after treatment with 6-OHDA.

Given that inhibiting GSK3β blocked an increase in SIRT2 activity, we next tested whether SIRT2 interacts with GSK3β by coimmunoprecipitation (Figures 3A,B). Our results indicate that GSK3β and SIRT2 may interact in cells.

To test whether GSK3β directly phosphorylates SIRT2, we carried out an *in vitro* kinase assay. We purified GST-SIRT2 from *E. coli* BL21. Because it has been reported that SIRT2 is a phosphorylation substrate for CDK5, we used CDK5/p25 (p25 continues to activate CDK5) as a positive control. We found that GSK3β could phosphorylate SIRT2 (Figure 3C). To determine which sites are phosphorylated by GSK3β, we carried out an *in vitro* kinase assay followed by SDS-PAGE and Coomassie blue staining to detect phosphorylated SIRT2, which was later evaluated by MS (Supplementary Figure S1). Finally, we identified three sites that could be phosphorylated by GSK3β (Figure 3D). To validate the MS results, we mutated these three sites to alanine residues to mimic dephosphorylation. The mutants were purified from *E. coli* and subjected to the kinase assay. We found that each mutant decreased the phosphorylation of SIRT2 by GSK3β (Figure 3E). To further test whether the interaction between GSK3β and SIRT2 changes under 6-OHDA stress, we performed a coimmunoprecipitation assay. It was shown that the interaction between GSK3β and SIRT2 was increased under 6-OHDA treatment (Figure 3F). In conclusion, we identified S327, S331 and S335 as SIRT2 phosphorylation sites targeted by GSK3β.

### SIRT2 Phosphorylation-Resistant Mutants Can Alleviate SIRT2 Toxicity in SH-SY5Y Cells

**Figure 4:**
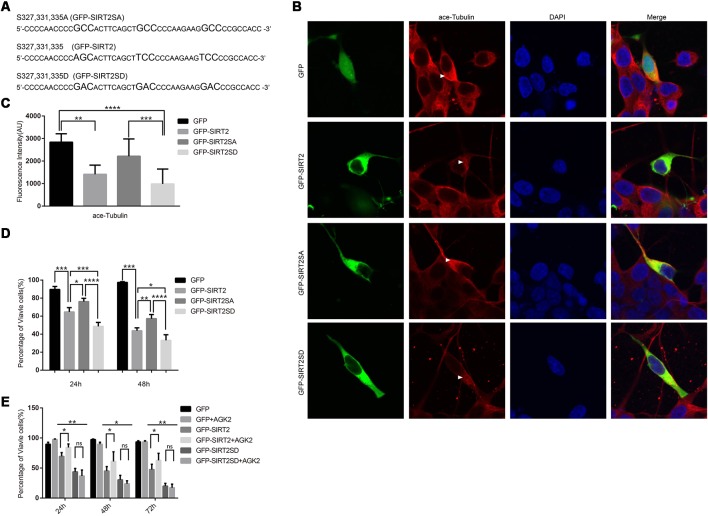
A SIRT2 phosphorylation mimic enhanced SIRT2 cytotoxicity. **(A)** GFP-SIRT2 is the wild-type plasmid DNA sequence, GFP-SIRT2SA is the dephosphorylation mimic sequence, and GFP-SIRT2SD is the phosphorylation mimic sequence. **(B)** Fluorescence images showing ace-tubulin expression in cells transfected with GFP, GFP-SIRT2, GFP-SIRT2SA or GFP-SIRT2SD; the fluorescence intensity of ace-tubulin is shown in red (arrowheads). **(C)** A histogram representing the fluorescence intensity of ace-tubulin immunofluorescence in cells transfected with GFP, GFP-SIRT2, GFP-SIRT2SA or GFP-SIRT2SD (**p < 0.01, ***p < 0.001, ****p < 0.0001, one-way ANOVA followed by Tukey’s *post hoc* test). **(D)** A histogram representing the percentage of viable cells. The percentage of viable cells in GFP-, GFP-SIRT2-, GFP-SIRT2SA- or GFP-SIRT2SD-transfected cells was calculated after 24 and 48 h (**p < 0.01, ***p < 0.001, ****p < 0.0001, two-way ANOVA followed by Tukey’s *post hoc* test). **(E)** A histogram representing the percentage of viable cells. AGK2 was added after the cells were transfected for 24 h. The percentage of viable cells was calculated 24 h, 48 h and 72 h after the addition of AGK2 (*p < 0.05, **p < 0.01, two-way ANOVA followed by Tukey’s *post hoc* test). The values are expressed as the mean ± SD, *n* ≥ 3 for each group (*p < 0.05, **p < 0.01, ***p < 0.005, ns, not statistically significant; two-tailed Student’s *t*-test).

To further prove that the phosphorylation of SIRT2 by GSK3β does participate in 6-OHDA-induced SH-SY5Y cell death and that overactivated SIRT2 aggravates PD (Outeiro et al., 2007), we constructed two mutants, GFP-SIRT2SA and GFP-SIRT2-SD, to mimic dephosphorylation and phosphorylation, respectively (Figure 4A). Given that ace-tubulin is the substrate of SIRT2 (North et al., 2003), we measured the fluorescence intensity of ace-tubulin after the individual overexpression of GFP, GFP-SIRT2, GFP-SIRT2SA or GFP-SIRT2SD, and we found that the mutant that mimicked phosphorylation further decreased the acetylation of α-tubulin, while the mutant that mimicked dephosphorylation did not (Figures 4B,C). This result indicates that the phosphorylation-resistant mutant (GFP-SIRT2SA) blocks SIRT2 deacetylation activity. As we showed that GSK3β regulates the phosphorylation of SIRT2 and that the protective effect of blocking GSK3β in the PD cell model occurs through decreased SIRT2 phosphorylation, we next transfected SH-SY5Y cells with the GFP, GFP-SIRT2, GFP-SIRT2SA, or GFP-SIRT2SD plasmids for 24 h or 48 h. Cell viability was measured by live cell counting, and the data showed a notable increase in the viability of the cells overexpressing GFP-SIRT2SA (the GFP-SIRT2-overexpressing cells relative to the GFP-SIRT2SA-overexpressing cells, *P < 0.05, **P < 0.01), whereas there was no increase or decrease in the viability of the cells overexpressing GFP-SIRT2SD (the GFP-SIRT2-overexpressing cells relative to the GFP-SIRT2SD-overexpressing cells, *P < 0.05, ***P < 0.001; Figure 4D). To assess whether AGK2 is able to block phospho-SIRT2 toxicity, we overexpressed the indicated plasmids and found that AGK2 was able to block GFP-SIRT2 toxicity but not GFP-SIRT2SD toxicity (Figure 4E).

These data indicate that the phosphorylation of SIRT2 by GSK3β at S327, S331 and S335 accelerates cell death. All of the data suggest that the dephosphorylation of S327, S331, and S335 decreases the activity of SIRT2, which could slow the cell death induced by SIRT2 (GFP-SIRT2) overexpression in SH-SY5Y cells.

## Discussion

SIRT2 appears to play a detrimental role in neurological disorders such as PD (Outeiro et al., 2007; Chen et al., 2015), Huntington’s disease (Luthi-Carter et al., 2010) and ischemic stroke (Krey et al., 2015; Xie et al., 2017; She et al., 2018; Wu et al., 2018), and it is important to understand how SIRT2 is regulated since we know little about it. In the present study, we found that SIRT2 phosphorylation was increased in a cellular model of PD and that this change was mediated by the activation of GSK3β. We further identified three phosphorylation sites in SIRT2 that are targeted by GSK3β and found that a genetic mutant that mimics the dephosphorylation of SIRT2 can alleviate cell death induced by SIRT2 overexpression. Several types of evidence support this finding: (i) using Western blot analysis, we found that the phosphorylation of endogenous SIRT2 in SH-SY5Y cells is increased after treatment with 6-OHDA; (ii) SB216763, an inhibitor of GSK3β, reduced the phosphorylation of endogenous SIRT2; (iii) an *in vitro* kinase assay showed that SIRT2 is phosphorylated by GSK3β. Phosphoproteomic analysis of SIRT2 revealed that three particular sites are responsible for this phosphorylation; and (iv) genetic mutants that mimic the phosphorylation or phosphorylation resistance of the three phosphorylation sites aggravates or alleviates SIRT2-induced cell death in SH-SY5Y cells, respectively.

In this study, we first determined the subcellular location of SIRT2 under 6-OHDA treatment and found that it remains in the cytoplasm. SIRT2 regularly localizes to the cytoplasm (North et al., 2003), and it has also been reported to localize to the nucleus during the G2/M phase (Dryden et al., 2003; North et al., 2003). This prompted us to search for an upstream regulator of SIRT2 in the cytoplasm. A previous study showed that the acetylation of GSK3β increases in pathological cardiac hypertrophy and that SIRT2 binds to, deacetylates and activates GSK3β (Sarikhani et al., 2018). SIRT2 may also act as an upstream regulator of GSK3β to modulate the differentiation of dopaminergic neurons (Szegö et al., 2017). Here, we proposed that the increase in SIRT2 phosphorylation mediated by GSK3β affects SIRT2 catalytic activity as well. To obtain the data presented here, we used a mutant that mimics the dephosphorylation of SIRT2 (GFP-SIRT2SA) and found that it does not decrease α-tubulin acetylation, which is the opposite of what is observed with the mutant that mimics the phosphorylation of SIRT2 (GFP-SIRT2SD). Compared to GFP-SIRT2, GFP-SIRT2SA significantly increased the survival of SH-SY5Y cells. This result coincides with that of a previous study (Outeiro et al., 2007). We showed that SIRT2 phosphorylation increases after exposure to 6-OHDA, and we speculated that SIRT2 phosphorylation accelerates cell death. As expected, cell death worsened in the GFP-SIRT2SD group. Thus, the mutant that mimics the dephosphorylation of SIRT2 (GFP-SIRT2SA) can lessen the cell death induced by SIRT2 overexpression by decreasing SIRT2 deacetylase activity. All of these data suggest that there are complex regulatory relationships between SIRT2 and GSK3β.

We found that AGK2 could not inhibit GFP-SIRT2SD toxicity. This suggests that the phosphorylation sites we found here may be important for the ligand docking of AGK2. In previous studies, decreased enzymatic activity was observed in several C-terminal alanine substitution mutations, such as mutations at S327 or S331 and S335 (Nahhas et al., 2007). Interestingly, S331A and S335A individually did not affect SIRT2 enzymatic activity, but double substitutions elicited a 44% reduction in activity (Nahhas et al., 2007). These reports are consistent with our finding that GFP-SIRT2SA reduces SIRT2 deacetylation activity. In contrast, the phosphorylation of S331 or a phospho-mimicking mutant of this site also inhibits the catalytic activity of SIRT2 (Pandithage et al., 2008). This report differs from our finding that GFP-SIRT2SD does not reduce SIRT2 deacetylation activity. This may be due to the simultaneous substitution of three serine residues in SIRT2. A preferred site for ligand binding of AGK2 is the “C-pocket” with a hydrogen-bonding pattern that mimics the action of nicotinamide, a known inhibitor of sirtuins (Outeiro et al., 2007). Since S327, S331 and S335 are located outside of the “C-pocket,” these serine residues may affect the SIRT2 enzymatic activity by altering the conformation of the protein. In fact, nicotinamide blocks the activity of the phospho-mimicking mutant GST-SIRT-S331D (Pandithage et al., 2008), suggesting that AGK2 also inhibits the activity of GST-SIRT2-S331D. Similarly, we observed that AGK2 does not inhibit GFP-SIRT2SD toxicity, which may be because three simultaneous substitutions alter the conformation of the “C pocket,” resulting in the failure of AGK2 docking. Our results indicate that S327, S331 and S335 can be simultaneously phosphorylated by GSK3β *in vitro*, and we are not sure whether this modification occurs *in vivo*. More experiments are needed to study this phenomenon.

Interestingly, it has been reported that CDK5 phosphorylates SIRT2 at S331 (Pandithage et al., 2008), S331 and S335 (Zhang et al., 2018), and we found that S327, S331 and S335 are phosphorylated by GSK3β. Furthermore, the phosphorylation of SIRT2 was found to be stronger after the coapplication of CDK5 and GSK3β (data not shown). These results indicate that there may be crosstalk between the GSK3β and CDK5 pathways. The data we present here suggest that phosphorylation-resistant mutants inhibit the catalytic activity of SIRT2. Nevertheless, phosphorylation by CDK5 inhibits the catalytic activity of SIRT2 (Pandithage et al., 2008). S331 is a common phosphorylation site, and the opposite effect may rely on other phosphorylation sites. Thus, targeting CDK5 and GSK3β at the same time may be a potential direction for the development of therapeutic strategies for PD. One possible approach is to block CDK5 and GSK3β simultaneously with one or two inhibitors. Using this strategy, we can obtain inactivated SIRT2 that is unphosphorylated at S327, S331 and S335. This may be the most effective strategy to suppress SIRT2 expression (Nahhas et al., 2007). There are many inhibitors currently in clinical trials that can block CDK5 and GSK3β simultaneously, such as AZD5438 and brain permeable AZD1080. Roscovitine and CHIR-99021, which are inhibitors of CDK5 and GSK3β, respectively, can also be used together. However, it is worth noting that the activities of CDK5 and GSK3β are related and that crosstalk depends on age (Engmann and Giese, 2009). In young mice, an increase in p25-induced CDK5 activity inhibits GSK3β activity by enhancing inhibitory phosphorylation at Ser-9 of GSK3β (Plattner et al., 2006). However, in older mice, GSK3β activity is enhanced in p25 transgenic mice (Plattner et al., 2006; Wen et al., 2008). Thus, the reaction of young and aged mice to the simultaneous targeting of CDK5 and GSK3β may be different.

As aging is considered a major risk factor for the development of PD, the pathways involved in aging may provide targets for therapeutic interventions for PD. SIRT2 is a member of the sirtuin family of proteins, which participate in a variety of cellular functions and play a role in aging (Maxwell et al., 2011). Outeiro et al. (2007) found that the inhibition of SIRT2 rescues α-synuclein toxicity and modifies inclusion morphology in a cellular model of PD (Dillin and Kelly, 2007; Garske et al., 2007). Later, de Oliveira et al. (2017) showed that α-synuclein is deacetylated by SIRT2 and that α-synuclein acetylation is a key regulatory mechanism governing α-synuclein aggregation and toxicity. Here, we further characterized SIRT2, showing that its activity is activated by its phosphorylation by GSK3β in a cellular model of PD. Based on the above conclusions, we elucidated a complete GSK3β-SIRT2-α-synuclein signaling cascade that participates in the development of PD. Because of the importance of α-synuclein in PD, we believe this cascade may play a vital role in PD. We may find treatments that inhibit the progression of PD by targeting this cascade.

Taken together, the data presented here highlight a previously unexplored cellular pathway that might underlie the impairment of dopaminergic neurons in PD, further underscoring the potential of the GSK3β-SIRT2-α-synuclein signaling cascade as a viable target pathway for neuroprotective therapies.

## Author Contributions

XW designed the experiments. SL and ZZ performed the enzyme activity assays and the biochemical analysis of the SH-SY5Y cells. LZ, SM and SLi analyzed the data. SL and ZZ wrote the manuscript with help from XW. All authors read and approved the final manuscript.

## Conflict of Interest Statement

The authors declare that the research was conducted in the absence of any commercial or financial relationships that could be construed as a potential conflict of interest.

## Supplementary Material

FIGURE S1 Phosphopeptide identification by mass spectrometry (MS). **(A)** GST-SIRT2 was purified from BL21 *E. coli*, and an *in vitro* kinase assay was performed with or without GSK3β. The samples were separated *via* SDS-PAGE, and then the gel was visualized with Coomassie blue staining. The band in the red rectangle was subjected to MS. **(B)** The serine residues in red are the predicted phosphorylated sites.
