# Supplementary figures and images for: Inhibition of SIRT2 by Targeting GSK3β-Mediated Phosphorylation Alleviates SIRT2 Toxicity in SH-SY5Y Cells
Source: Front Cell Neurosci. 2019 Apr 24;13:148. doi: 10.3389/fncel.2019.00148 (PMC6492038; doi:10.3389/fncel.2019.00148)

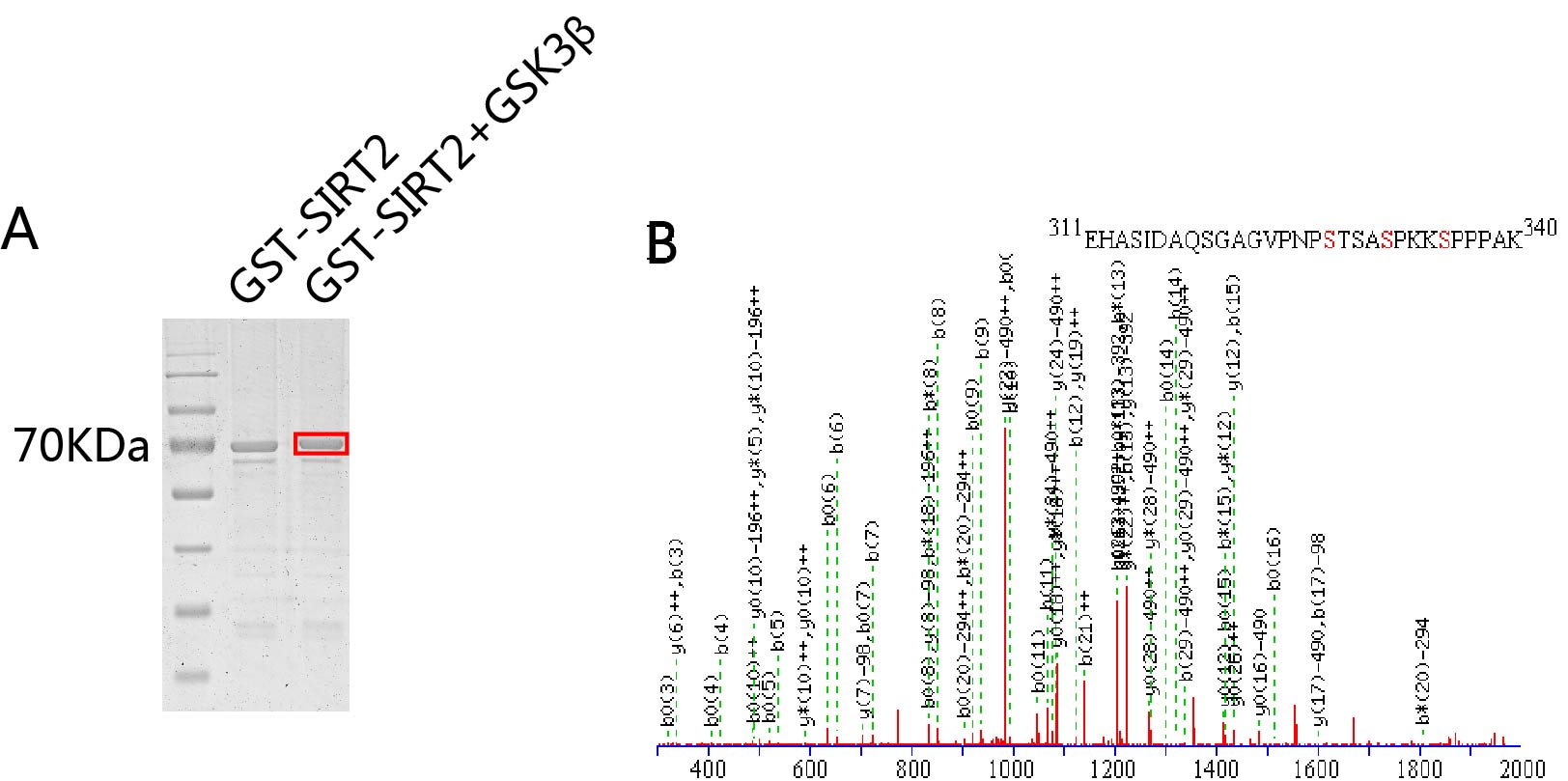

Supplement: FIGURE S1 — Phosphopeptide identification by mass spectrometry (MS). (A) GST-SIRT2 was purified from BL21 E. coli, and an in vitro kinase assay was performed with or without GSK3β. The samples were separated via SDS-PAGE, and then the gel was visualized with Coomassie blue staining. The band in the red rectangle was subjected to MS. (B) The serine residues in red are the predicted phosphorylated sites. [file Image_1.JPEG]
